# Key hub genes and pathways associated with HCV-related hepatocellular carcinoma as potential diagnostic biomarkers

**DOI:** 10.1016/j.jgeb.2026.100721

**Published:** 2026-06-11

**Authors:** Elham Karimi, Niloufar Sadat Kalaki, Mahnaz Shavandi, Fargol Mahlouji, Abed El Hasan Haidar, Aria Abedi Asl, Fahimeh Safarnezhad Tameshkel, Farhad Zamani, Mohammad Hadi Karbalaie Niya

**Affiliations:** a Department of Medical Genetics, School of Medicine, Tehran University of Medical Sciences, Tehran, Iran; b Department of Medical Genetics, School of Medicine, Shahid Beheshti University of Medical Sciences, Tehran, Iran; c Department of Stem Cell and Regenerative Medicine, Institute of Medical Biotechnology, National Institute of Genetic Engineering and Biotechnology, Tehran, Iran; d Department of Internal Medicine, School of Medicine, Iran University of Medical Sciences, Tehran, Iran; e Gastrointestinal and Liver Diseases Research Center, Iran University of Medical Sciences, Tehran, Iran; f Bowen Medical Center, Bowen, QLD, Australia; g Department of Virology, School of Medicine, Iran University of Medical Sciences, Tehran, Iran

**Keywords:** Hepatocellular carcinoma, Hepatitis C virus, Bioinformatics, Differentially expressed gene

## Abstract

**Background:**

Hepatitis C virus (HCV)-related hepatocellular carcinoma (HCC) remains a major global health challenge, with high morbidity and mortality despite recent therapeutic advances. Early detection and identification of reliable molecular biomarkers are essential to improve patient outcomes. Therefore, the present study aimed to investigate key hub genes and pathways associated with HCV-related HCC as potential diagnostic biomarkers.

**Methods:**

The datasets GSE69715 and GSE62232 were obtained from the Gene Expression Omnibus (GEO) database. Differentially expressed genes (DEGs) were recognized according to an adjusted *p*-value and a log fold change (logFC). The GEO2R tool facilitated the identification of common DEGs across the two datasets. Pathways were explored using the Kyoto Encyclopedia of Genes and Genomes (KEGG) and Gene Ontology (GO) databases. Furthermore, protein-protein interactions (PPIs) were assessed through Cytoscape. The target genes were confirmed through a GEPIA analysis.

**Results:**

A total of 421 common DEGs were identified, and 80 hub genes were subsequently determined through GEO and PPI network analyses, respectively. The GO and KEGG pathways analysis presented DEGs were enhanced in metabolic pathways, cellular components, extracellular exosome, detoxification of copper ion and monooxygenase activity. The GEPIA analysis indicated a notable variation in the expression levels of four specific genes —CDKN2A, CDK1, CCNB1, and TOP2A—when comparing normal samples to tumor samples.

**Conclusion:**

The present study discovered novel genes by expression variation in HCV-related hepatocellular carcinoma development. These findings suggest that CDKN2A, CDK1, CCNB1, and TOP2A are promising candidates for diagnostic biomarkers and present a valuable opportunity for the early identification of HCV-HCC, which could lead to improved treatment outcomes.

## Highlights

• There are novel genes by expression variation in HCV-related hepatocellular carcinoma development.
• CDKN2A, CDK1, CCNB1, and TOP2A can potentially be utilized as diagnostic biomarkers.

## 1 Introduction

Liver cancer, known medically as hepatic cancer, is the third most significant contributor to cancer-related mortality.^1^ Although there are several types of liver cancer, hepatocellular carcinoma (HCC) is the most typical, prevalent, and deadly form globally.^2^ The most widespread variant of primary liver cancer is hepatocellular carcinoma (LIHC), characterized by a low 5-year survival rate.^3^ Less common types of liver cancer include intrahepatic cholangiocarcinoma, which begins in the bile ducts within the liver, and angiosarcoma, which arises from the liver's blood vessels.^4^ Liver cancer frequently develops in individuals with chronic liver conditions like cirrhosis or hepatitis B or C infections. HCC is recognized as the most commonly diagnosed condition, primary liver cancer and is a major factor in cancer-related fatalities across the globe.

The presence of hepatitis C virus (HCV) infection is a significant factor influencing the likelihood of developing HCC, which remains a pressing health concern internationally. Research indicates that around 55% to 85% of individuals with HCV infection will develop chronic hepatitis C, with 20% to 30% of those suffering from chronic liver disease potentially advancing to liver failure or cirrhosis.^5^ Persistent HCV infection can result in prolonged liver inflammation, fibrosis, cirrhosis, and eventually the development of HCC.^6^ The development of HCV-induced HCC involves complex interactions between viral factors and the host's immune responses. Early diagnosis in HCC patients is crucial for improving their prognosis. Abnormal gene expression and alterations play an essential role in HCC progress and enhancement. The quick advancement of bioinformatics, in conjunction with the rise of high-throughput methods like microarray and next-generation sequencing (NGS), has been essential to progress in this sector.^7^

Comprehensive datasets, encompassing DNA copy number evaluations, genomic sequencing, and protein array systems, are currently being archived in public repositories like the Gene Expression Omnibus (GEO) and Oncomine IVD (In Vitro Diagnostic). These repositories play a critical role in the identification of differentially expressed genes (DEGs) and the exploration of the molecular mechanisms that contribute to carcinogenesis in HCC.^6^ Studies have revealed substantial mutations in three genes, which are pertinent to the prognosis of HCC. Specifically, the EXO (Exonuclease 1) gene, VCAN (Versican) gene, and KIT (Receptor Tyrosine Kinase Type III) gene have been identified as possible markers for biological assessment for HCV-induced HCC.^8^ Over expression of DNA Topoisomerase 2α (TOP2A),^9^ CCNB1, CDC20, and CENPF genes was a common event in hepatocarcinogenesis.^3^ The significant overexpression of CCNB1, KIF20A, and HMMR is recognized as a biomarker for both the diagnosis and therapeutic strategies for HCC associated with HCV.^6^, ^10^

Recent studies have highlighted the role of genetic polymorphisms and immune-related genes in the progression of HCC. For instance, Bakr et al.^11^ demonstrated that genetic variations significantly influence disease susceptibility and progression through inflammatory and metabolic pathways. Similarly, Bakr et al.^12^ reported that interleukin-21 gene polymorphism is associated with increased risk of hepatocellular carcinoma in HCV-infected patients. These findings emphasize the importance of identifying molecular biomarkers and genetic signatures involved in HCC development.

Therefore, the primary objective of this study was to systematically identify and validate key hub genes and signaling pathways involved in HCV-related HCC using integrated bioinformatics approaches, with the aim of discovering potential diagnostic biomarkers and therapeutic targets.

## 2 Methods and materials

### 2.1 Microarray data

The datasets GSE69715 (including 37 patients with HCV-HCC and 66 healthy individuals) and GSE62232 (including 9 patients with HCV-HCC and 10 healthy individuals) utilizing via the GPL570 Affymetrix Human Genome U133 Plus 2.0 Array yielded the data. Both datasets were acquired from the Gene Expression Omnibus (GEO) database (http://www.ncbi.nlm.nih.gov/geo/), which hosts a wide range of freely accessible microarray gene expression datasets. The datasets were chosen according to the following criteria: (1) inclusion of samples from Human HCV-HCC, and (2) the existence of a case-control group.

### 2.2 The commonly differentially expressed genes (DEGs)

A comparative analysis of common DEGs (differentially expressed genes) between patient samples and normal specimens was performed utilizing GEO2R (https://www.ncbi.nlm.nih.gov/geo/geo2r/) The GEO2R tool was used to identify differentially expressed genes (DEGs) between patient and control samples. Differentially Expressed Genes (DEGs) were identified by applying criteria of adjusted *P* values below 0.05, with logFC (fold change) values equal to or greater than 1, and logFC values equal to or less than −1, which were subsequently chosen for network construction. Furthermore, the Bioinformatics and Evolutionary Genomics Venn diagram tool (http://bioinformatics.psb.ugent.be/webtools/Venn/) was employed to create Venn diagrams for the comparison of upregulated and downregulated DEGs.

### 2.3 Functional enrichment analysis

The Gene Ontology (GO) (http://www.geneontology.org) is a prominent framework in bioinformatics, extensively employed for the large-scale annotation of genes and their products, thereby becoming a fundamental element in research activities. The analysis focused on three biological facets: biological process (BP), molecular function (MF), and cellular component (CC). The Kyoto Encyclopedia of Genes and Genomes (KEGG) (https://www.kegg.jp/) provides a practical database resource that aids in polymer experiments and genome sequencing. To determine the mechanisms by which a gene is enhanced, molecular information from macromolecular datasets is analyzed. The DAVID database (https://david.ncifcrf.gov/) was used to perform GO term analysis on these differentially expressed genes (DEGs), with a significance level set at *P* < 0.05. This method facilitated the identification of significant biological functions and pathways associated with the DEGs, elucidating their potential contributions to HCV-related HCC.

### 2.4 PPI network construction and performance analysis

In order to identify hub genes from their protein-protein interaction (PPI) network, differentially expressed genes (DEGs) were submitted to the STRING server (https://string-db.org; version 11.5). The PPI network was analyzed using centrality parameters such as degree, betweenness, and closeness. Cytoscape (version 3.6.0) was employed to construct the PPI network by importing the output file from STRING, which enabled the analysis of key genes within the network. Hub genes were detected using Cytoscape based on centrality parameters, including degree, betweenness, and closeness. For further analysis, we will focus on the ten hub genes that demonstrate the greatest degree of connectivity in the PPI network.

### 2.5 Verification and validation of hub genes using TCGA

To confirm the dependability and prognostic significance of the hub genes discovered from GEO datasets, RNA sequencing data (HTSeq-FPKM) along with the relevant clinical information of patients with hepatocellular carcinoma (HCC) were sourced from The Cancer Genome Atlas (TCGA) database (https://portal.gdc.cancer.gov/). The Gene Expression Profiling Interactive Analysis (GEPIA, http://gepia.cancer-pku.cn/) platform, which merges TCGA and GTEx data, was utilized to compare gene expression levels between tumor and normal liver tissues and to create Kaplan–Meier survival curves for the analysis of overall survival. Statistical significance was established with a threshold of *p* < 0.05.

### 2.6 Co-expression network validation

RNA-seq data from the TCGA-LIHC dataset were analyzed to assess the co-expression of four key genes (CCNB1, CDK1, CDKN2A, and TOP2A). Pearson correlation coefficients were computed between the hub genes and all other protein-coding genes. Strong correlations (|r| > 0.5) were depicted in a heatmap generated using heatmap.2 (gplots package), featuring hub genes as columns and associated co-expressed genes as rows. The heatmap colors range from blue to red, indicating negative to positive correlations, which underscores the pivotal role of hub genes within the TCGA co-expression network.

### 2.7 Immune infiltration analysis

We examined the relationship between hub genes (CDK1, CCNB1, CDKN2A, TOP2A) and tumor-infiltrating immune cells utilizing the TIMER2.0 database. For every hub gene, we retrieved the correlation of its expression with the presence of different immune cell types across TCGA datasets. We filtered the results to find statistically significant correlations (adjusted *p*-value <0.05) and chose the top 5 immune cell types exhibiting the highest absolute Spearman correlation (rho) for each gene. Heatmaps were created to illustrate the correlation patterns using R (packages: tidyr, dplyr, pheatmap).

### 2.8 Drug-gene interaction analysis

In order to investigate possible therapeutic targets, the four identified hub genes (CDKN2A, CDK1, CCNB1, TOP2A) were examined in the Drug-Gene Interaction Database (DGIdb) and DrugBank to find drugs with known interactions. The identified drugs were sorted according to their mechanism of action (e.g., inhibitor) and their approval status (Approved / Investigational).

### 2.9 Statistical analysis

We calculated the values of DEGs from the GEO DataSets, with adjusted *P*-values below 0.05 considered statistically significant. For GO and KEGG enrichment analyses, a P-value threshold of less than 0.05 was used to determine significance. The Analysis-Box Plots module of GEPIA, with settings of *p*-values <0.05, |log2FC| > 1 and matching TCGA normal to GTEx data, was used to explore the expression levels of genes associated with HCC.

## 3 Results

### 3.1 Common DEGs

**Fig. 1:**
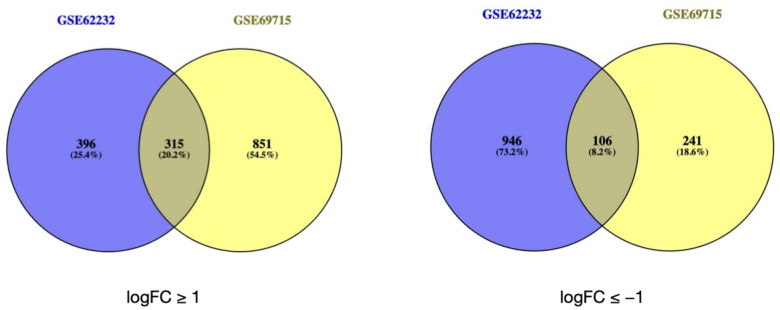
The identification of 421 common differentially expressed genes (DEGs) between the GSE69715 and GSE62232 datasets was conducted using Venn diagram software. Distinct colors were utilized to represent each dataset. The analysis employed the Bioinformatics and Evolutionary Genomics Venn tool, focusing on a log fold change (logFC) threshold of ≤ −1 and ≥ 1.

The datasets GSE69715 and GSE62232 were chosen from the GEO database. Venn diagram software was then used to identify the common DEGs across the two datasets (Fig. 1). The analysis revealed 421 common DEGs, comprising 315 upregulated DEGs and 106 downregulated DEGs. A comprehensive list of these 421 common DEGs (Fig. 1) is available in Supplementary Table 1.

### 3.2 GO and KEGG pathway enrichment the common DEGs

**Fig. 2:**
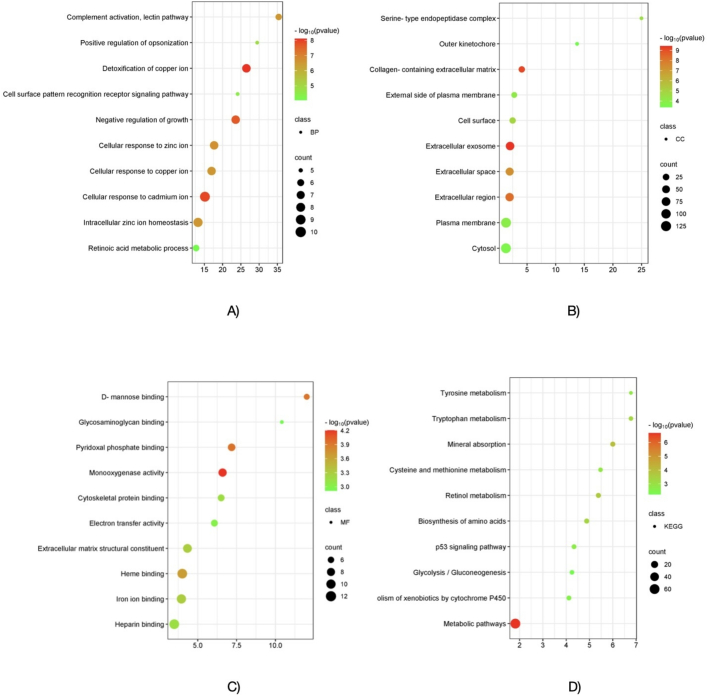
An analysis conducted by DAVID bioinformatics has identified the ten most significant gene ontology processes related to various expressed proteins, which are classified into: (A) Biological Processes (BP), (B) Cellular Compartments (CC), (C) Molecular Functions (MF), and (D) KEGG pathways.

Analyses of GO annotation and KEGG pathway enrichment were performed using the DAVID and Enrich databases, respectively. Fig. 2 shows the top ten enriched GO terms for each GO category (BP, CC, MF) and the top ten enriched KEGG pathways for hub genes. According to the findings, the GO biological process analysis indicated that the 421 DEGs were significantly enriched in the detoxification of copper ions, cellular responses to cadmium ions, negative regulation of growth, and cellular responses to zinc ions. The four most significantly enriched cellular components were identified as the extracellular exosome, collagen-containing extracellular matrix, extracellular region, and extracellular space. For the GO molecular function analysis, the four most significantly enriched terms included monooxygenase activity, pyridoxal phosphate binding, D-mannose binding, and heme binding. The pathways that exhibited significant enrichment for these 421 DEGs were metabolic pathways, mineral absorption, retinol metabolism, and the biosynthesis of amino acids. As illustrated in Fig. 2, the enrichment results indicate that metabolic pathways and extracellular components play a central role in HCV-related HCC progression, suggesting their involvement in tumor microenvironment remodeling and metabolic dysregulation.

### 3.3 PPI network and hub genes

**Fig. 3:**
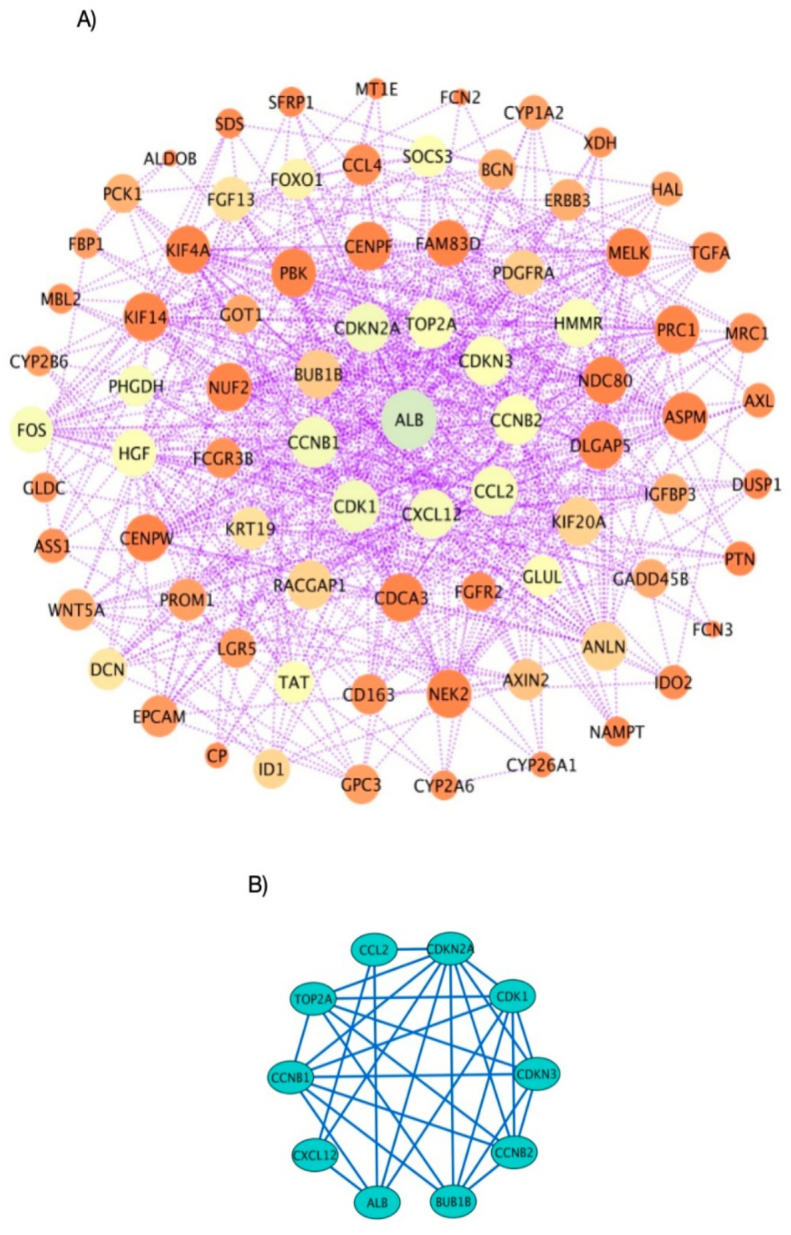
The examination of Protein–Protein Interactions (PPI) is conducted. Node size and color reflect the degree and betweenness centrality, respectively. A) The PPI network showcases the hub genes identified from the shared differentially expressed genes in GSE69715 and GSE62232. (B) The top ten hub genes with the greatest degree of connectivity in the PPI network are presented.

The construction of a protein-protein interaction (PPI) network was based on the STRING database. Out of the 421 identified DEGs, only 313 genes were successfully mapped into the STRING database and included in the PPI network. This discrepancy is due to the exclusion of genes lacking sufficient interaction data or failing to meet the minimum confidence score required for network construction. Subsequently, Cytoscape software was employed to visualize the PPI network (Fig. 3A). Analyzing these PPI networks allows for the identification of significant molecular interactions that may affect disease progression. A total of 421 commonly expressed differentially expressed genes (DEGs) were identified from the GEO datasets, with 313 genes being successfully mapped and incorporated into the protein-protein interaction (PPI) network, leading to a clustering coefficient of 0.337 and a network centralization of 0.203. Hub genes were prioritized according to their degree, closeness, and betweenness centrality, as detailed in Supplementary Table 2. By utilizing the STRING server with the designated parameters, key hubs were identified, revealing common genes among the top 80 nodes, which exhibited a clustering coefficient of 0.660 and a network centralization of 0.356 (see Supplementary Table 3). The ten genes with the highest connectivity degree—albumin (ALB), cyclin dependent kinase 1 (CDK1), cyclin B1 (CCNB1), cyclin B2 (CCNB2), C—C motif chemokine ligand 2 (CCL2), cyclin dependent kinase inhibitor 3 (CDKN3), topoisomerase II alpha (TOP2 A), C-X-C motif chemokine ligand 12 (CXCL12), BUB1 mitotic checkpoint serine/threonine kinase B (BUB1B), and cyclin dependent kinase inhibitor 2 A (CDKN2A)—were identified through Cytoscape (refer to Fig. 3B). The ten hub genes exhibiting the highest connectivity were selected for further investigation.

### 3.4 Validation of hub genes in the TCGA- LIHC cohort

**Fig. 4:**
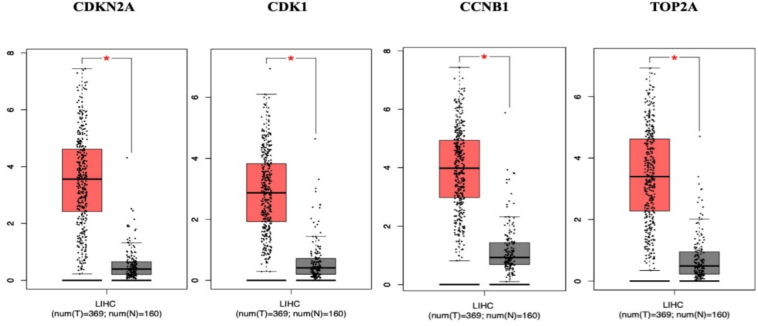
The Box plots of the hub genes, CDKN2A, CDK1, CCNB1 and TOP2A. The analysis revealed a significant difference between the samples classified as normal and those identified as tumors (*P < 0.05).

The results from GEPIA, utilizing GEO datasets, indicate that certain hub genes hold significant prognostic relevance in HCV-HCC. Out of the ten hub genes examined, several displayed elevated expression levels in HCV-HCC samples when compared to normal samples, highlighting their potential as biomarkers (Fig. 4). Four of these genes—cyclin-dependent kinase inhibitor 2 A (CDKN2A), cyclin-dependent kinase 1 (CDK1), cyclin B1 (CCNB1), and DNA topoisomerase II alpha (TOP2A)—demonstrated considerable upregulation in tumor tissues in contrast to normal liver samples (*P* < 0.05 for all genes).

**Fig. 5:**
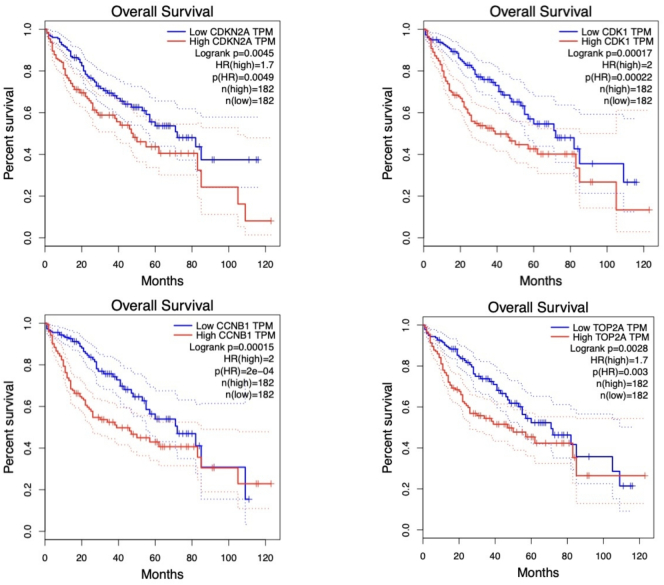
The comprehensive survival analysis of the hub genes indicated a notable correlation between the expression levels of CDKN2A, CDK1, CCNB1, and TOP2A. and overall survival outcomes. HCC patients with increased expression of these hub genes were found to have a significantly diminished overall survival time (*P* < 0.05).

**Fig. 6:**
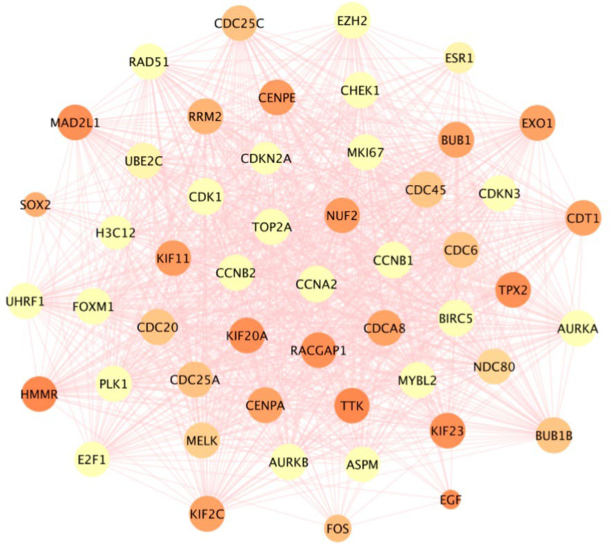
Displays the Protein–Protein Interaction (PPI) network of hub genes confirmed in the TCGA-LIHC cohort. The size and color of the nodes represent the degree and betweenness centrality, respectively. This network showcases the connections between the validated hub genes, emphasizing highly interconnected nodes that could act as crucial regulators in the progression of HCC.

To confirm these results in a separate dataset, the expression levels and prognostic significance of the same four hub genes were evaluated using RNA-seq data from the TCGA-LIHC cohort through GEPIA. In line with the GEO findings, all four genes showed a significant increase in expression in tumor tissues (*p* < 0.001, Fig. 5A–D). Additionally, Kaplan–Meier survival analysis verified that greater expression of these genes correlated with reduced overall survival in HCC patients (*p* < 0.05, Fig. 5E–H). The findings illustrate the consistency and prognostic importance of the hub genes across various datasets and platforms. To delve deeper into the interactions among the hub genes confirmed in the TCGA-LIHC cohort, a PPI network was created utilizing the STRING database. This network consisted of XX nodes and XX edges, with the size and color of the nodes indicating degree and betweenness centrality, respectively (Fig. 6). Nodes that are highly interconnected may signify crucial regulators of HCC development and possible targets for additional functional investigations. The full list of hub genes confirmed in the TCGA-LIHC cohort can be found in Supplementary Table 4.

### 3.5 Validation of hub gene co-expression networks in TCGA

**Fig. 7:**
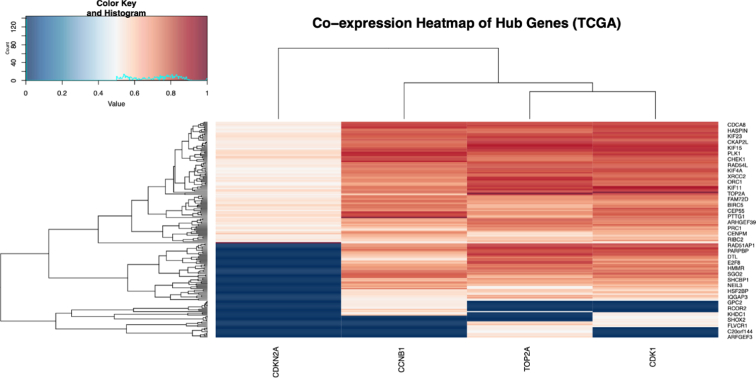
Analysis of Pearson correlation among the four hub genes (CCNB1, CDK1, CDKN2A, TOP2A) and all protein-coding genes in TCGA HCC samples. Significant correlations (|r| > 0.5, p < 0.05) are indicated. Red and blue denote positive and negative correlations, respectively, emphasizing the pivotal roles of these hub genes within the HCC co-expression network. (For interpretation of the references to color in this figure legend, the reader is referred to the web version of this article.)

To assess the integration of the four selected hub genes (CCNB1, CDK1, CDKN2A, TOP2A) in pathways related to HCC, a co-expression analysis was conducted using RNA-seq data from TCGA. Pearson correlations were calculated between the hub genes and all other protein-coding genes. Only strong correlations (|r| > 0.5, *p* < 0.05) were taken into account. The heatmap presented in Fig. 7 demonstrates that these hub genes exhibit substantial centrality within the co-expression network, indicating their potential regulatory significance in HCC.

### 3.6 Immune infiltration analysis

**Fig. 8:**
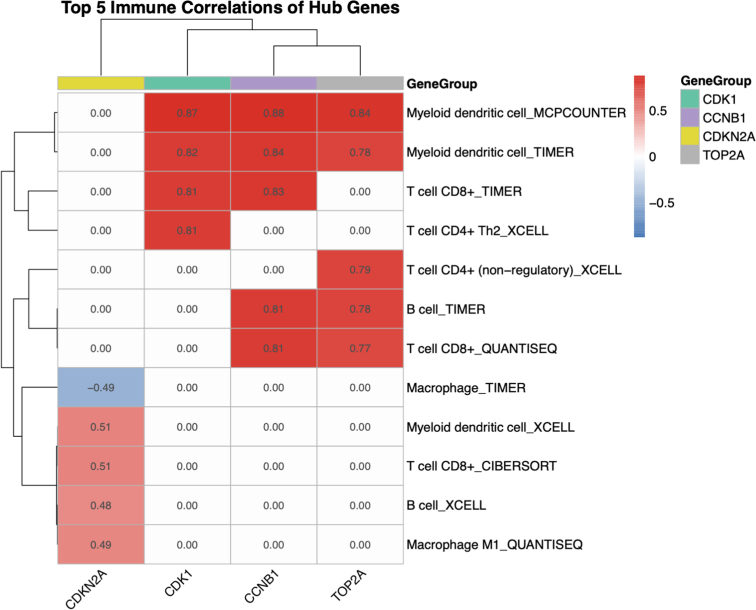
Primary immune associations of key genes in liver hepatocellular carcinoma (HCC). The rows reflect types of immune cells, while the columns represent key genes. The color gradient illustrates Spearman correlation coefficients (blue = negative, white = neutral, red = positive). The values in the cells denote the correlation values. (For interpretation of the references to color in this figure legend, the reader is referred to the web version of this article.)

To investigate the connection between hub genes and the tumor immune microenvironment, we examined the relationship between the expression levels of CDK1, CCNB1, CDKN2A, and TOP2A and the presence of various immune cell types across TCGA cancers utilizing TIMER2.0. For each gene, we identified the top five immune cell types that displayed the strongest statistically significant correlations (adjusted *p*-value <0.05). The analysis demonstrated that: CDK1 and CCNB1 showed strong positive relations with myeloid dendritic cells, CD8+ T cells, and CD4+ T cells, indicating that these genes might impact both antigen presentation and adaptive immune responses. CDKN2A was positively associated with myeloid dendritic cells, CD8+ T cells, Macrophage M1, and B cells, emphasizing its possible role in regulating both innate and adaptive immunity. TOP2A exhibited similar trends to CDK1 and CCNB1, with significant links to dendritic cells, CD4+ T cells, CD8+ T cells, and B cells. In summary, these results suggest that the expression of hub genes is closely linked to the infiltration of critical immune cell populations, indicating a potential connection between these genes and the tumor immune environment (Fig. 8).

### 3.7 Drug-gene interaction analysis

A comprehensive list of drugs targeting the validated hub genes (CDKN2A, CDK1, CCNB1, and TOP2A) was sourced from the Drug-Gene Interaction Database (DGIdb) (see Table 1). Most of the drugs identified function as inhibitors of the specific products of these hub genes. Among the agents that target TOP2A, several compounds—such as doxorubicin hydrochloride, mitoxantrone hydrochloride, etoposide, teniposide, valrubicin, dexrazoxane, daunorubicin hydrochloride, etoposide phosphate, idarubicin, and amsacrine—are approved by the FDA for clinical application. Conversely, the majority of the compounds that target CDKN2A, CDK1, and CCNB1 are either investigational or experimental, highlighting their potential for future therapeutic development.

**Table 1:** Drug-gene interactions for HCV-HCC hub genes (from DGIdb).

Drug Name	Gene	Mechanism of Action	Approval Status
Doxorubicin hydrochloride	TOP2A	Inhibitor	Approved
Mitoxantrone hydrochloride			
Etoposide			
Teniposide			
Valrubicin			
Dexrazoxane			
Daunorubicin hydrochloride			
Etoposide phosphate			
Idarubicin			
Amsacrine			
RGB-286638	CDK1	Inhibitor	Investigational
Dinaciclib			
Roniciclib			
Milciclib			
AZD5438			
AT-7519			
Alvocidib			
Zotiraciclib			
Seliciclib			

## 4 Discussion

Despite advancements in the treatment of hepatocellular carcinoma (HCC), its rates of incidence and mortality remain elevated. By analyzing the GSE69715 and GSE62232 datasets, we identified 421 common differentially expressed genes, many of which are associated with the regulation of cell division. Significantly, the overexpression of CDKN2A, CDK1, CCNB1, and TOP2A is linked to a poor prognosis in cases of HCV-related HCC. Furthermore, validation with RNA-seq data from the independent TCGA-LIHC cohort confirmed that these four hub genes were consistently upregulated in tumor tissues and correlated with decreased overall survival. This reproducibility across different platforms highlights the reliability of these hub genes as potential prognostic biomarkers in HCV-related HCC.

Our findings are consistent with previous bioinformatics studies that identified CDK1, CCNB1, and TOP2A as critical regulators of cell cycle progression in HCC.^13^, ^14^ However, the novelty of the present study lies in the integration of multiple GEO datasets, validation using TCGA data, and the incorporation of immune infiltration and drug–gene interaction analyses. This comprehensive approach provides a more robust understanding of the molecular mechanisms underlying HCV-related HCC.

According to the literature, overexpression of these genes is related to cancer development. The over expression of CDKN2C (p18) has also been detected in HCV-HCC. CDKN2C is vital in the control of the cell cycle, by inhibiting the activity of CDK4 and CDK6, which results in the halting of the cell cycle during the G1 phase. Overexpression of CDKN2C in HCV-HCC suggests its involvement in the pathogenesis of HCC by contributing to cell cycle dysregulation and potentially promoting tumorigenesis.^15^ Significant up-regulation of CDKN2C and CDKN2A mRNAs in HCC has been documented. Microarray analyses have consistently shown elevated levels of CDKN2C mRNA in HCC, while CDKN2A mRNA is up-regulated in pancreatic tumors.^16^ High expression of CDKN2A is recognized as a distinct prognostic factor for colorectal cancer (CRC).^17^ Various tumors exhibit CDKN2A expression, which positively correlates with prognosis and immune cell infiltration, showing high levels in most cases. Generally, CDKN2A expression is linked to negative cancer outcomes, suggesting a potential connection to tumor immunity.^18^ In the past few years, there has been a notable increase in research demonstrating the link between topoisomerase and cancer, particularly IIA topological isomers, are recognized as important therapeutic targets for drugs designed to treat cancer and microbial diseases.^19^ TOP2A, expressed by the TOP2A gene, is a DNA topoisomerase participating in transcription and replication processes by altering DNA's topological structure. Its overexpression often correlates with increased tumor proliferation and aggressiveness.^20^ Prior research has established that abnormal expression of TOP2A is present in several cancer subtypes, including HCC, colon, gastric breast, prostate cancers,ovarian^7^ and non-small cell lung cancer (NSCLC).^21^ high TOP2A expression has been linked to a higher tumor grade, increased Ki67 index (an indicator of cellular proliferation), and a higher rate of distant metastasis.^20^ The effects of TOP2A gene knockout and overexpression can either inhibit or stimulate the proliferation, metastasis, and invasion of HCC cells in vitro and in vivo. TOP2A is responsible for activating the cell cycle transition from G2 to M phase by preventing CHK1 phosphorylation and promoting epithelial-mesenchymal transformation. Therefore, TOP2A significantly influences the proliferation, migration, invasion, and epithelial-mesenchymal transition of HCC.^22^ The combination of the findings indicates that greater expression of TOP2A corresponds with more evident malignant biological behavior in tumors and a worse prognosis for patients. This points to the potential role of TOP2A as an independent prognostic factor in HCC, where high levels of expression in tumor tissue may be indicative of a poor prognosis for patients.^23^ CDK1, a critical kinase in the control of the cycle of mammalian cells, has been determined to be a key gene within the network with notable connectivity. The target genes of CDK1 have been previously established, highlighting their roles in transcriptional processes, chromosome segregation, cell morphogenesis, DNA replication, and the stability of the genome.^24^ The association between CDK1 and another oncogenic molecule is vital and has a substantial impact on metastasis.^25^ It has been verified that the inhibition of CCNB1/CDK1 results in the reactivation of p53 function, suggesting that CCNB1 might play a role in tumorigenesis by disrupting p53 activity.^26^ Abnormal activation of CDK1 can lead to uncontrolled cell proliferation, a hallmark of cancer.^27^ In HCV-induced HCC, CDK1 is often found to be overexpressed, contributing to the malignant transformation of hepatocytes.^28^ CCNB1 (Cyclin B1) is a crucial protein involved in the regulation of the cell cycle, particularly in the transition from the G2 phase to mitosis. Overexpression of CCNB1 has been observed in HCV-induced HCC. This overexpression can lead to uncontrolled cell proliferation, contributing to tumor growth and progression.^3^ The knockdown of CCNB1 resulted in a significant reduction in the proliferation, invasion, and migration of HCC cells in both in vitro and in vivo settings. Consequently, CCNB1 may serve as a valuable prognostic biomarker and a potential therapeutic target within the tumor microenvironment of HCC.^29^

We acknowledge the limitation of not including experimental validation. However, the scope of this study was to perform an initial in silico screening and prioritization of potential diagnostic markers for HCV-HCC. Our aim was to provide a computational basis for future experimental work. Given the availability of high-throughput datasets and the robustness of the bioinformatics methods applied, we believe our findings offer valuable preliminary insights. Further experimental validation is encouraged in future studies to confirm these results.

The analysis of immune infiltration indicated that hub genes, such as CDK1, CCNB1, CDKN2A, and TOP2A, have strong associations with key immune cell types in HCC. CDK1 and CCNB1 demonstrated significant positive relationships with myeloid dendritic cells, CD8+ T cells, and CD4+ T cells, suggesting their potential role in enhancing antigen presentation and supporting adaptive immune responses. CDKN2A was also linked to B cells and M1 macrophages, highlighting its participation in both innate and adaptive immunity. TOP2A showed a similar correlation pattern to that of CDK1 and CCNB1. These results indicate that the altered expression of hub genes may not only aid in tumor progression but also influence the tumor immune microenvironment. Consequently, these genes could be potential biomarkers for evaluating immune infiltration and serve as targets for improving immunotherapy approaches in HCC. Additionally, TOP2A is a target of FDA-approved therapeutic drugs. Since we identified these genes through notable differences in HCC-HCV patients, they are promising candidates as diagnostic biomarkers.

Nevertheless, to validate our findings and explore a potential direction, we can understand from other studies that there are experimentally confirmed significant levels of increased expression in CDKN2A, CDK1, CCNB1, and TOP2A, indicating their remarkable diagnostic value.^13^, ^14^, ^30^ Furthermore, TOP2A is targeted by therapeutic drugs that have received FDA approval.^13^ Given that we identified these genes through significant differentiation in HCC-HCV patients, they hold considerable promise as diagnostic biomarkers.

Importantly, the identification of these hub genes may contribute to improved diagnostic strategies and personalized therapeutic approaches, particularly through targeting cell cycle dysregulation and tumor immune interactions in HCV-related HCC patients.

### 4.1 Limitation

We acknowledge that this study is purely bioinformatic and lacks experimental validation. Therefore, the identified hub genes and pathways should be considered preliminary. Future in vitro and in vivo studies, such as RT-qPCR and western blotting in HCC cell lines and patient tissues, are necessary to confirm our findings and elucidate the functional roles of these genes in HCV-related HCC pathogenesis.

## 5 Conclusion

In conclusion, this study identified key hub genes and pathways associated with HCV-related HCC through integrated bioinformatics analysis. Notably, CDKN2A, CDK1, CCNB1, and TOP2A were highlighted as potential diagnostic biomarkers and therapeutic targets. These findings may contribute to improving early detection, guiding treatment strategies, and enhancing clinical outcomes in patients with HCV-related HCC. The common feature among the genes CDKN2A**,** CDK1**,** CCNB1**,** and TOP2A is being an essential factor in cell cycle control and cell division; any dysregulation may result in unchecked cell proliferation, which can contribute to the development of tumors.

## CRediT authorship contribution statement

**Elham Karimi:** Writing – original draft, Software, Methodology, Investigation, Data curation. **Niloufar Sadat Kalaki:** Writing – original draft, Supervision, Resources, Methodology, Data curation. **Mahnaz Shavandi:** Writing – original draft, Validation, Methodology, Investigation, Formal analysis. **Fargol Mahlouji:** Visualization, Software, Investigation, Formal analysis. **Abed El Hasan Haidar:** Validation, Methodology, Formal analysis, Data curation. **Aria Abedi Asl:** Writing – original draft, Supervision, Software, Methodology, Investigation. **Fahimeh Safarnezhad Tameshkel:** Writing – review & editing, Supervision, Project administration, Formal analysis, Conceptualization. **Farhad Zamani:** Visualization, Supervision, Resources, Formal analysis. **Mohammad Hadi Karbalaie Niya:** Writing – review & editing, Validation, Project administration, Funding acquisition, Conceptualization.

## Funding Acknowledgment

All Authors thanks for supports of this project which granted by the 10.13039/100012021Iran University of Medical Sciences, by the grant code: 1404-2-105-34221.

## Declaration of competing interest

The authors declare that they have no known competing financial interests or personal relationships that could have appeared to influence the work reported in this paper.
