# Supplementary material for: Key hub genes and pathways associated with HCV-related hepatocellular carcinoma as potential diagnostic biomarkers
Source: J Genet Eng Biotechnol. 2026 Jun 11;24(3):100721. doi: 10.1016/j.jgeb.2026.100721 (PMC13276315; doi:10.1016/j.jgeb.2026.100721)
Supplement: Supplementary file 1 — Supplementary material [file mmc1.docx]

**Supplementary Tables**

**Supplementary Table 1. The common DEGs were screened according to adjusted P- values < 0.05 and logFC ≥ 1 and logFC ≤ −1.**

| CLEC4G, FCN3, GPM6A, CLEC1B, COLEC10, IDO2, OIT3, FCN2, ECM1, CLEC4M, MARCO, RSPO3, HHIP, CXCL14, TFPI2, CRHBP, CD5L, KAZN, EPHB1, FAM65C, STAB2, PITPNM3, DACH1, PRKAR2B, ADAMTS13, ASS1, ANGPTL6, IL13RA2, CCBE1, APOA5, ST6GAL2, HAMP, CHST4, SFRP1, LIFR, CYP2B6, PAMR1, CLDN10, PLAC8, LINC01093, CETP, MASP1, PHGDH, SPG20, FREM2, CYP2A6, ALDOB, PCDH9, ZGPAT///LIME1, MFSD2A, SRD5A1, P3H2, FCGR2B, EPB41L4B, MAN1C1, PELI2, ATOH8, TUBE1, GCH1, GBA3, NPY1R, GADD45B, TACSTD2, IGFBP3, TIMD4, HAND2-AS1, ANXA3, PROM1, GSTZ1, TTC36, MYO10, KRTCAP3, GPR180, AVPR1A, KMO, GLS2, EDNRB, ID2, DNASE1L3, FEZ1, ADAMTS1, FNIP2, RDH16, TCF21, BBOX1, ADRA1A, BCKDHB, CXCL12, PLPP3, HAO2, TMEM27, SLC41A2, CYP39A1, APOF, FAM149A, MOGAT2, SLC7A2, CDH19, SLC25A47, FBP1, PDE7B, STEAP4, GHR, S100A8, FAM134B, C4orf19, HGF, LY6E, ANGPTL1, TENM1, GLYAT, SRPX, CLRN3, ETNPPL, SOCS2, CAND2, CYP1A2, TUSC1, SLC38A4, RBMS3, MPC1, ZG16, PTPRS, TAT, ADH4, ITGA9, MIR675///H19, BCO2, NEBL, IL1RAP, PRSS8, GRAMD1C, EIF4E3, GYS2, SLC22A1, ABHD2, CDA, ADGRG7, DSEL, OLFML3, PEMT, SYNPO2, BDH2, CYP2B7P, C8orf4, CTH, PEG3, TSPYL5, FOXO1, VNN 1.00, MRC1, MS4A6A, AZGP1P1///AZGP1, COLEC11, GREM2, ANK3, CYP4V2, ADGRG6, MT1F, CCDC71L, UPP2, DCN, ACSL1, GGT5, SHBG, AKR1D1, AMIGO2, FCGR3B, PDE4B, ADORA3, DPT, SLC19A3, AFM, GSPT2, HS3ST3B1, SEMA6D, DSE, KCND3, PCK1, SLCO1B3, GAREM1, PLSCR4, TDO2, PNP, PRG4, AMDHD1, PTN, HOTS, ETS2, CNTN3, ALB, BASP1, SYTL5, ARRB1, ZFPM2, C11orf96, DUSP5, XDH, SPIDR, MCC, AXL, MXRA5, TGFA, SEMA5A, SLC8A1, MT1G, NAMPT, RND3, MT1X, MGLL, MT1E, LEPROT///LEPR, MS4A7, SLCO4C1, PON3, MT1HL1, BGN, THRSP, MT1M, CD163, PLGLB1///PLGLB2, FOS, SERPINA4, AKR7A3, SDS, PDGFRA, CEBPD, CDC37L1, HPS5, LINC01558, PLD1, CYP26A1, MT2A, NNMT, C7, FOXF1, ITGB8, GLT1D1, SLC27A2, HAL, CD1D, CNTN4, MT1H, TSPAN12, SKAP1, ACOT12, MUM1L1, GLDC, SAMD5, KYNU, BHMT, CCL4, TBX15, ANTXR2, CA2, FOSB, INMT, NR4A3, C1orf162, RAB27A, VSIG4, ABI3BP, RGS2, HPGD, HABP2, SLC4A4, CYFIP2, SOCS3, WDR72, CRISPLD2, C9, SAA2-SAA4///SAA4, SLC1A1, ENO3, ALDH8A1, ADH1C, ASPN, ADH1B, ADAMTSL3, ID1, FGFR2, CP, PTGIS, EMILIN1, MMRN1, KRT19, GOT1, LOC101928916///NNMT, CITED2, MBL2, IGHM, AKAP12, NR4A2, SPATA18, SERPINA5, CYP2C18, DUSP1, EPCAM, CCL2, CPM, TSPAN13, ZEB2, FETUB, MIR3682, SLC2A9,RBP1, SPP2, NRG1, G6PC, SMIM24, SOX6, TMEM45A, FAM26F, SGCB, JCHAIN, CAP2, RACGAP1, PSMD4, FLVCR1, ASPM, CDKN2C, DLG5, HMMR, TOP2A, GBAP1, CDKN2B, UBAP2L, PRUNE1, PRC1, CCNB1, CDK1, GOLPH3L, SATB2, MSH5-SAPCD1///SAPCD1///MSH5, NEK2, MTR, ANLN, TAPT1-AS1, STXBP6, CENPF, CENPW, TPR, TBCE, SMYD2, CDKN3, CDKN2A, PBK, GSTA4, PDZK1, NDC80, KIF14, KIF20A, LGALS8, C1orf112, FAM72A///FAM72D///FAM72B///FAM72C, BUB1B, MELK, DLGAP5, SQLE, PLPPR1, MTMR11, FIGNL1, ERBB3, FDPS, FGF13, SLC25A43, CDCA3, MIR452///MIR224///GABRE, NEK3, NMRAL1P1, MAP3K13, CCNB2, TP53I3, CDC42BPA, TDGF1P3///TDGF1, EDIL3, LOC401068, NUF2, KIF4A, EFNA4, CEMIP, FAM83D, COX20, GLUL, FAM169A, GPC3, ZIC2, MSH5-SAPCD1///SAPCD1, LOC101930578///MRPS31P5///THSD1, GRK3, HOXA3, PHYHIPL, SFN, CRNDE, RUSC1-AS1, PRAP1, RBM24, IRX3, RHEB, RNF43, EML6, EPS8L3, FRMD3, SPARCL1, WNT5A, SP5, LOC102724689, LGR5, ASPSCR1, SPINK1, DUXAP10, KCNJ5, DUT, LINC01021, MGC32805, PIR-FIGF///FIGF, HOXA13, ZNRF3, AXIN2, SLC22A11, SMPX. |
| --- |

**Supplementary Table 2. Network Specifications of DEGs compared with Hub genes.**

| **Network** | **Network diameter** | **Network centralization** | **Clustering coefficient** | **Characteristic path on length** |
| --- | --- | --- | --- | --- |
| 313 nodes | 10 | 0.203 | 0.337 | 3.527 |
| 80 nodes | 4 | 0.356 | 0.660 | 2.222 |

**Supplementary Table 3. 80 Hub genes from the PPI network of differential expressed genes (number of nodes: 80, clustering coefficient: 0.660, network centralization: 0.356)**

| CYP26A1, SOCS3, FGFR2, GADD45B, CYP2A6, CENPW, FAM83D, CCNB2, GPC3, CDKN3, DUSP1, CDCA3, AXL, CDK1, KIF4A, PHGDH, NEK2, BUB1B, CYP2B6, IDO2, CCL4, PBK, CDKN2A, KIF14, FOXO1, MELK, ALDOB, GLUL, PRC1, CENPF, CCL2, FBP1, TOP2A, MRC1, ID1, HMMR, CD163, GOT1, NUF2, MBL2, ASPM, CXCL12, RACGAP1, FGF13, XDH, PROM1, FOS, WNT5A, CP, TAT, IGFBP3, KRT19, KIF20A, MT1E, PTN, CYP1A2, DCN, HGF, NAMPT, HAL, CCNB1, SFRP1, PDGFRA, PCK1, ASS1, EPCAM, SDS, LGR5, TGFA, ERBB3, FCN3, ALB, GLDC, AXIN2, FCN2, ANLN, DLGAP5, FCGR3B, BGN, NDC80. |
| --- |

**Supplementary Table 4. 51 Hub genes from the PPI network of differentially expressed genes (number of nodes: 51, clustering coefficient: 0.934, network centralization: 0.148)**

| CDC25A, MELK, RAD51, EGF, AURKB, HMMR, PLK1, NUF2, FOS, ASPM, BUB1, TPX2, E2F1, RACGAP1, TTK, TOP2A, CDC45, CDK1, CDT1, CDC25C, ESR1, FOXM1, CDKN2A, UHRF1, CDC6, AURKA, CCNB2, MYBL2, CHEK1, CDKN3, CCNB1, H3C12, KIF23, MAD2L1, KIF11, CDC20, NDC80, MKI67, CCNA2, EZH2, EXO1, SOX2, KIF20A, CENPE, UBE2C, BIRC5, RRM2, CENPA, CDCA8, BUB1B, KIF2C. |
| --- |
